# *Plasmodium knowlesi* Infection in Humans, Cambodia, 2007–2010

**DOI:** 10.3201/eid1710.110355

**Published:** 2011-10

**Authors:** Nimol Khim, Sovannaroth Siv, Saorin Kim, Tara Mueller, Erna Fleischmann, Balbir Singh, Paul Cliff Simon Divis, Nicolas Steenkeste, Linda Duval, Christiane Bouchier, Socheat Duong, Frederic Ariey, Didier Ménard

**Affiliations:** Author affiliations: Pasteur Institute of Cambodia, Phnom Penh, Cambodia (N. Khim, S. Kim, F. Ariey, D. Ménard);; National Center for Parasitology, Entomology, and Malaria Control, Phnom Penh (S. Siv, S. Duong);; University of Munich, Munich, Germany (T. Mueller, E. Fleischmann);; University Malaysia Sarawak, Kuching, Malaysia (B. Singh, P.C.S. Divis);; Fondation Mérieux, Phnom Penh (N. Steenkeste);; Centre International de Recherches Médicales de Franceville, Franceville, Gabon (L. Duval);; Institut Pasteur, Paris, France (C. Bouchier)

**Keywords:** Plasmodium knowlesi, simian malaria, epidemiology, Southeast Asia, malaria, parasites, vector-borne infections, zoonoses, Cambodia, dispatch

## Abstract

Two cases of *Plasmodium knowlesi* infection in humans were identified in Cambodia by 3 molecular detection assays and sequencing. This finding confirms the widespread distribution of *P. knowlesi* malaria in humans in Southeast Asia. Further wide-scale studies are required to assess the public health relevance of this zoonotic malaria parasite.

In Cambodia, malaria ranks among the leading causes of illness and death. Mostly affecting the ≈3 million persons (23% of Cambodia’s population) who live near forested areas, malaria remains an occupational disease in specific high-risk groups, such as forestry workers and migrant populations who have come into forested areas. However, for the past decade, the number of reported malaria cases has generally decreased but in a sawtooth pattern of periodic increases (*1*).

Four of the 5 *Plasmodium* species known to cause malaria in humans have already been described in Cambodia (*2**,**3*). *P. falciparum* remains the most frequent cause of malaria (83,777 cases in 2009, prevalence of 70%) (*1*). However, distributions of *Plasmodium* species are changing, with a particularly substantial increase of *P. vivax* malaria cases, from 4,105 (8%) cases in 2000 to 6,250 (25%) in 2009. In several areas of low transmission, the proportion of *P. vivax* infections has increased up to 50% (*2*). This trend is probably related to various effective control strategies implemented in Cambodia against *P. falciparum* malaria.

No studies in humans (*3*) and monkeys in Cambodia have identified the simian malaria parasite, *P. knowlesi*, which is causing human disease in some other countries in Southeast Asia (*4*). *P. knowlesi* parasites were not detected in blood samples collected during 2004–2007 for 138 monkeys (102 *Macaca fascicularis* monkeys; 13 *M. leonina* monkeys; 20 *Hyobates pileatus* monkeys; 2 *Presbytis cristata* monkeys; and 1 *Pongo pygmaeus* monkey) by using PCR (*cytb* and *cox1* genes) (L. Duval, unpub. data). Because these animals were confiscated by Wildlife Alliance rescue patrols from illegal traders, the locations where they were trapped in Cambodia are unknown.

We undertook a cross-sectional prospective study in 3 sites in Cambodia (Figure, panel A) (*5*). The main objective of this study was to develop evidence to guide the management of malaria parasite–negative persons with acute febrile illness and to determine whether such persons were infected with *P. knowlesi*.

**Figure:**
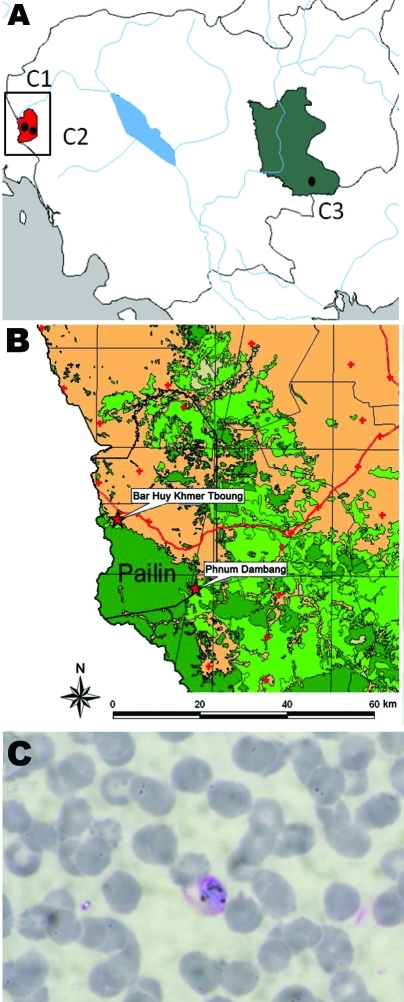
A) The 3 sites involved in the epidemiologic study of *Plasmodium knowlesi* malaria, Cambodia, 2007–2010. B) Villages in Pailin Province in which the 2 *P. knowlesi* malaria case-patients lived. C) Thin blood film slide of 1 PCR-confirmed *P. knowlesi* single infection (case C2611). Original magnification ×1,000.

## The Study

We enrolled all patients >7 years of age with acute (<8 days) febrile illness in selected outpatient clinics, where they had been tested for malaria by using rapid diagnostic test (CareStart, Access Bio Inc, Somerset, NJ, USA). A subset of nonfebrile patients were used as controls; patients in a critical clinical condition were excluded. After we obtained informed consent, each patient’s history was taken, a physical examination was conducted, and blood and throat swab samples were collected. The defined test battery, including the following pathogens, was performed by using a PCR/sequencing approach: *Plasmodium* spp., *Leptospira* spp., *Rickettsia* spp. (including *Orientia tsutsugamushi*), dengue viruses, Japanese encephalitis virus, and influenza viruses. HIV infection and tuberculosis were not evaluated. DNA and RNA from the erythrocyte pellet or throat swab were extracted by using the QIAamp kit (QIAGEN, Hilden, Germany), according to the manufacturer’s instructions. Malaria parasites were detected by using a *Plasmodium* spp.–specific nested PCR based on the *cytb* gene followed by sequencing (*6*).

During December 2007–December 2010, a total of 1,475 patients were enrolled (621 in Soun Kouma; 650 in Ou Chra, Pailin Province; and 204 in Snoul, Kratié Province), comprising 1,193 febrile and 282 nonfebrile persons. For 564 (38.2%) patients, no pathogens were detected. A total of 754 patients (51.1%; 676 cases, 78 controls) were infected with malaria parasites, and the distribution of *Plasmodium* spp. was as follows: *P. vivax*, 51.6%; *P. falciparum*, 40.6%; *P. vivax/P. falciparum*, 7.4%; *P. knowlesi*, 0.3%; and *P. ovale*, 0.1%.

We detected *P. knowlesi* infections in 2 patients from Ou Chra health center in Pailin Province (Figure, panel B). The first patient was a 41-year-old man from Borhuytbong village. He came in April 2010 to the health center with fever (38.5°C), chills, and headache. Rapid diagnostic test and microscopy were negative for malaria; PCR/sequencing was positive for *P. knowlesi*. No other pathogen was found. The patient was originally from Thailand and reported that he had not returned to Thailand since getting married in Cambodia in 1989. He reported self-treatment with mefloquine (Lariam [Roche, Basel, Switzerland]) and was cured in 3 days. He further added that he used to hunt and spent most of his time in forests around Pailin, where long-tailed macaques, the natural hosts of *P. knowlesi* (*7*), are usually found.

The second patient was a 40-year-old Khmer man from Phnomdambang village. He came in September 2010 to the health center because of chills and headache. No fever (36°C) was detected by the medical staff, but the patient reported a history of fever and had self-treated with acetaminophen. Results of rapid diagnostic test were negative, but microscopy results were positive (33 parasites/µL) (Figure, panel C). PCR amplification followed by sequencing confirmed *P. knowlesi* infection. No other pathogen was found. The patient reported having lived in this same village since 1995, and he frequently went to the forests around Pailin for hunting and collecting valued wood.

Both patients’ *P. knowlesi* infections were confirmed by additional PCR amplification and sequencing of the *ldh*, *tufA*, and *cox* genes (*8*) at the Genomic Platform, Institut Pasteur, Paris, France. Blood spots on filter paper were also sent blinded to the Malaria Research Centre, Faculty of Medicine and Health Sciences, University Malaysia, Sarawak, Malaysia, where they were identified as *P. knowlesi* single infections by real-time PCR (*9*) and by nested PCR with the primers Pmk8 and Pmkr9 (*10*) and PkF1140 and PkR1550 (*11*). The nucleotide sequence determined in this study has been deposited in the GenBank database and assigned accession nos. JF419317–JF419323.

## Conclusions

Our findings confirm that *P. knowlesi* infections occur in humans in Cambodia, thereby increasing the number of countries in Southeast Asia with cases in humans (*10*). However, further wide-scale studies are required to assess the prevalence and distribution of *P. knowlesi* malaria cases in humans and monkeys. Such studies would enable an assessment of the public health relevance of this zoonotic malaria parasite and characterization of *P. knowlesi* malaria epidemiology in this region. They would be particularly useful in determining whether this simian species has been imported from neighboring countries by humans or whether *P. knowlesi* parasites are already circulating and are transmitted from monkey reservoir hosts to humans. Moreover, to address this issue, development of new tools, such as specific serologic markers, is urgently needed, in addition to molecular biology methods.
